# Multidrug- and Extensively Drug-Resistant Tuberculosis, Germany

**DOI:** 10.3201/eid1411.080729

**Published:** 2008-11

**Authors:** Barbara Eker, Johannes Ortmann, Giovanni B. Migliori, Giovanni Sotgiu, Ralf Muetterlein, Rosella Centis, Harald Hoffmann, Detlef Kirsten, Tom Schaberg, Sabine Ruesch-Gerdes, Christoph Lange, for the German TBNET Group

**Affiliations:** Research Center Borstel, Borstel, Germany (B. Eker, S. Ruesch-Gerdes, C. Lange); Karl-Hansen-Clinic, Bad Lippspringe, Germany (J. Ortmann); World Health Organization Collaborating Center for TB and Lung Diseases, Tradate, Italy (G.B. Migliori, R. Centis); Sassari University, Sassari, Italy (G. Sotgiu); Regional Hospital, Pasberg, Germany (R. Muetterlein); Asklepios-Hospital, Gauting, Germany (H. Hoffmann); Hospital Großhansdorf, Großhansdorf, Germany (D. Kirsten); Deakoness Hospital, Rotenburg, Germany (T. Schaberg)

**Keywords:** tuberculosis, drug-resistance, MDR TB, XDR TB, linezolid, Germany, research

## Abstract

We evaluated risk factors and treatment outcomes associated with multidrug-resistant (MDR) and extensively drug-resistant (XDR) tuberculosis (TB) in Germany in 2004–2006. In 177 (4%) of 4,557 culture-positive TB cases, *Mycobacterium tuberculosis* isolates were identified as MDR TB; an additional 7 (0.15%) met criteria for XDR TB. Of these 184 patients, 148 (80%) were born in countries of the former Soviet Union. In patients with XDR TB, hospitalization was longer (mean ± SD 202 ± 130 vs. 123 ± 81 days; p = 0.015) and resistance to all first-line drugs was more frequent (36% vs. 86%; p = 0.013) than in patients with MDR TB. Seventy-four (40%) of these 184 patients received treatment with linezolid. Treatment success rates ranged from 59% for the entire cohort (59% for MDR TB and 57% for XDR TB) to 87% for those with a definitive outcome (n = 125; 89% for MDR TB and 80% for XDR TB). Extensive drug susceptibility testing and availability of second- and third-line drugs under inpatient management conditions permit relatively high treatment success rates in MDR- and XDR TB.

## Abstract 2

A survey by the TBNET in Germany shows that, under optimal circumstances, high sustained culture conversion rates can be achieved in advanced cases of MDR TB and XDR TB.

Tuberculosis (TB) is among the leading causes of death worldwide. The World Health Organization (WHO) estimates that 32% of the world population is infected with *Mycobacterium tuberculosis*, the causative agent of TB (*1*). There were an estimated 9.2 million new TB cases and 1.7 million deaths from TB in 2006 (*2*).

Drug resistance to isoniazid and rifampin, the 2 most potent first-line drugs for the treatment of TB (the definition for MDR), is increasing globally (*3*,*4*). Surveillance data indicate MDR TB is an emerging global problem, especially in countries of the former Soviet Union (FSU), Israel, and areas of the People’s Republic of China (*5*–*7*). Since active TB will develop in only a proportion of persons infected with *M. tuberculosis* directly after primary infection, the prevalence of MDR TB may still be underestimated. Furthermore, strains of *M. tuberculosis* that are resistant to second-line drugs are also emerging. In vitro drug resistance of *M. tuberculosis* to any fluoroquinolone and to at least one of the injectable drugs (capreomycin, kanamycin, or amikacin), in addition to isoniazid and rifampin resistance, is defined as XDR TB (*8*,*9*). Strains of XDR TB have now been isolated from patients in >45 nations worldwide, and they are associated with worse treatment outcomes than strains of MDR TB (*8*,*10*,*11*). Strains of XDR TB are increasingly seen in HIV-seropositive persons with TB in southern Africa, where these strains are passed by person-to person contact. XDR TB has become a serious problem for the health administrations in this region (*12*). In contrast, infections with XDR TB strains are rarely seen in Western Europe, mainly among the population of pretreated migrants from countries of the FSU (*13*).

Although the incidence of TB is steadily declining in Germany, numbers of cases with MDR TB strains are increasing. In 2006, of 3,501 TB cases in Germany for which resistance data were available, 78 (2.2%) were MDR TB (*14*); these cases mainly occurred among immigrants from countries with high prevalence of MDR TB (*14*,*15*).

TB surveillance data for Germany are reported annually by a national disease surveillance center, the Robert Koch Institute (*14*). However, data on MDR TB are only reported for in vitro first-line drug resistance against isoniazid, rifampin, ethambutol, pyrazinamide, and the injectable agent streptomycin. To ascertain risk factors associated with MDR and XDR TB and to evaluate treatment outcome in relation to level of drug resistance and level of care, we performed a retrospective survey among the network of hospitals participating in the Tuberculosis Network European Trials group (TBNET); these hospitals specialize in treating TB in Germany.

## Materials and Methods

Clinical outcomes (available from the original clinical records) were evaluated by attending physicians at hospitals specialized in the care of patients with TB in Germany; they completed a standard questionnaire for all patients with culture-confirmed isoniazid and rifampin drug-resistant *M. tuberculosis* hospitalized from January 1, 2004, through December 31, 2006. The survey included information on the patients’ age, gender, country of origin, HIV-seropositivity status, history of previous treatment, *M. tuberculosis* drug-resistance profile, treatment duration, and treatment outcome. Drug-susceptibility testing (DST) for first-line anti-TB drugs was performed by quality-assured laboratories. Isolates with resistances to anti-TB drugs were (re-)tested at one of the WHO’s Supranational Reference Laboratories in Borstel or Gauting (*16*). DST for second-line drugs (ethionamide, amikacin, capreomycin, p-aminosalicylic acid, cycloserine, kanamycin) or third-line drugs (linezolid) were exclusively performed in 1 of the 2 reference centers. The BACTEC MGIT 960 (Becton Dickinson Diagnostic Systems, Sparks, MD, USA) was used for DST of first-line drugs and BACTEC MGIT 960 or the proportion method on Lowenstein–Jensen medium, or both, was used for DST of second- and third-line drugs. XDR TB was defined as resistance to isoniazid and rifampin (MDR TB definition), a fluoroquinolone, and at least one of the injectable anti-TB drugs capreomycin, kanamycin, or amikacin (*17*). MDR TB cases with isolates resistant to all first-line drugs were defined as those resistant to isoniazid, rifampin, ethambutol, streptomycin and, when tested, pyrazinamide.

According to Laserson criteria, a patient was defined as “cured” when he or she had completed treatment according to the country protocol and had been consistently culture-negative (with at least 5 results) for the final 12 months of treatment; “treatment completed” when he or she had completed treatment according to the country protocol but did not meet the definition for cure or treatment failure or bacteriologic results were missing (i.e., <5 cultures were performed in the final 12 months of therapy) (*18*). Outcomes were compared by using the χ^2^ test or Fisher exact test (categorical variables) in cases achieving a final outcome (different from default, transferred out, or still on treatment), and by using the Kaplan-Meier curve where appropriate. Logistic regression analysis was performed. The following variables were included in the statistical analysis: country, gender, HIV seropositivity, immigrant status, previous TB treatment for >30 days, DST results (ethambutol, pyrazinamide, streptomycin, any fluoroquinolone, any injectable second-line drug), and resistance to all second-line drugs. A patient was considered HIV positive, when results of the HIV-antibody ELISA (once) and at least 1 confirmatory test (Western blot or nucleic acid amplification technique) were positive.

## Results

Among 4,557 culture-confirmed TB cases at 27 participating hospitals (representing 37% of all culture-confirmed cases in Germany in the 3-year period 2004–2006), 184 (4%) *M. tuberculosis* isolates were in vitro resistant at least to isoniazid and rifampin. They MDR TB isolates represented 65% of all MDR- and XDR TB cases diagnosed in Germany in the study period (*14*,*19*,*20*). Of these cases, 177 were MDR TB, and 7 were XDR TB.

Of the 184 study patients, 174 (95%) had *M. tuberculosis* isolates resistant to streptomycin, 119 (65%) to ethambutol, 103 (56%) to rifabutin, 79 (43%) to pyrazinamide, 23 (13%) to amikacin, 20 (11%) to a fluoroquinolone, 19 (10%) to capreomycin, 36 (19%) to ethionamide, 15 (8%) to para-aminosalicylic acid, 9 (5%) to cycloserine, 3 (2%) to kanamycin, and 1 (<1%) to linezolid. Demographic and clinical characteristics are described in Table 1 and the Appendix Table.

**Table 1:** Demographic and clinical characteristics of 184 patients with MDR TB and XDR TB, Germany*

Variables	MDR TB, n = 177	XDR TB, n = 7	p value	95% CI
Male gender, no. (%)	133 (75.1)	6 (85.7)	0.54	–0.37 to 0.17
Age, y, mean ± SD	37.7 ± 15.4	42.4 ± 11.9	0.42	–16.33 to 6.93
Country of birth, no. (%)				
Former Soviet Union	142 (80.2)	6 (85.7)	0.74	–0.32 to 0.22
Germany	11 (6.2)	–	–	–
Others	24 (13.6)	1 (14.3)	0.93	–0.27 to 0.25
HIV positive, no. (%)	7/142 (4.9)	0	0.54	0.01 to 0.08
Kind of TB, no. (%)				
Pulmonary TB	162 (91.5)	6 (85.7)	0.59	–0.2 to 0.32
Extrapulmonary TB	5 (2.9)	–	–	–
Pulmonary and extrapulmonary TB	10 (5.6)	1 (14.3)	0.29	–0.34 to 0.16
Days in hospital, mean ± SD	123.3 ± 81	202 ± 130	0.015†	–141.8 to –15.53
Previous TB treatment, no. (%)	94 (53)	6 (86)	0.08	–0.59 to 0.06
Resistance to all first-line drugs, no. (%)	64 (36)	6 (85.7)	0.008	–0.76 to –0.21
Resistance to fluoroquinolones, no. (%)	13/162 (8)	7 (100)	<0.001†	–0.96 to –0.87
Resistance to injectable second-line drugs, no. (%)	21/164 (12.8)	7 (100)	<0.001†	–0.92 to –0.83
Linezolid treatment, no. (%)	69 (39)	5 (71.4)	0.09	–0.66 to 0.02
Treatment outcome, no. (%)				
Cured	79 (44.6)	3 (42.8)	0.91	–0.35 to 0.39
Completed	26 (14.7)	1 (14.3)	1	–0.26 to 0.26
Successful treatment (cured + completed)	105 (59.3)	4 (57.1)	0.91	–0.35 to 0.39
Died	14 (7.9)	1 (14.3)	0.4	–0.32 to 0.18
Failure	1 (0.6)	–	–	–
Treatment failure (death or failure)	15 (8.4)	1 (14.3)	0.57	–0.32 to 0.2
Default	1 (0.6)	–	–	–
Transferred out	25 (14.1)	–	–	–
Uncertain outcome (default + transferred out)	26 (14.7)	–	–	–
Still on treatment	31 (17.5)	2 (28.6)	0.45	–0.44 to 0.22
Duration of therapy from beginning MDR treatment, mo, mean ± SD	18 ± 9	20 ± 5	0.56	–8.78 to 4.78
Sputum smear conversion, no. (%)	98 (55.4)	5 (71.4)	0.4	–0.5 to 0.18
Culture conversion, no. (%)	132 (74.6)	5 (71.4)	0.85	–0.31 to 0.37
Sputum smear conversion, d, mean ± SD	69.4 ± 76	129.8 ± 129.2	0.09	–132 to 11.2
Culture conversion, d, mean ± SD	81.3 ± 74.6	141 ± 99.7	0.08	–127.6 to 8.2

*MDR, multidrug-resistant; TB, tuberculosis; XDR, extensively drug-resistant; CI, confidence interval. †Significant result (p<0.05).

Forty-five (24%) patients with MDR TB strains were female (median age 28 years), and 139 (76%) were male (median age 39 years). HIV testing was performed for 142 (80%) of 177 patients with MDR TB and 4 (57%) of 7 patients with XDR TB. Seven patients with MDR TB (4.9%) and no patient with XDR TB tested positive for HIV-1. Notably, 148 (80.4%) of 184 patients with MDR TB were immigrants from the FSU (Appendix Figure).

Ninety-four (53%) patients with MDR TB and 6 (86%) patients with XDR TB had previously received anti-TB treatment for >1 month (p = 0.08). Of the 100 previously treated patients, 89% were immigrants from the FSU, 6% from other countries, and 5% were born in Germany. Only 1 of the 7 patients with XDR TB had previously received directly observed treatment. Strains from patients with XDR TB had a significantly higher probability to be resistant to all first-line drugs (isoniazid, rifampin, pyrazinamide, ethambutol) (6/7, 85.7% vs. 64/177, 36%; p = 0.08) than strains from other patients with MDR TB. The median time from the onset of treatment to conversion of smear microscopy and culture to negative results was 88 days (mean ± SD 129.8 ± 129.2 days) and 117 days (mean ± SD 141 ± 99.7 days), respectively, with XDR TB vs. 53.5 days (mean ± SD 69.4 ± 76.1 days) and 61.5 days (mean ± SD 81.3 ± 74.6 days), respectively, with MDR TB.

Of 177 patients with MDR TB, 14 (7.9%) died, one’s treatment failed (0.6%), 105 (59.3%) were treated successfully (6/105 underwent surgery), 31 (17.5%) were still receiving treatment, and 26 (14.7%) were lost to follow-up. Of 7 patients with XDR TB, 4 (57.1%) were treated successfully (1/6 underwent surgery), 2 (28.6%) were still receiving treatment, and 1 (14.3%) died.

The overall treatment success including all patients was 59.2% (59.3% for patients with MDR TB and 57.1% for patients with XDR TB). After the 26 patients lost to follow-up were removed from the analysis, the treatment success rate increased to 69% (69.5% for patients with MDR TB and 57.1% for patients with XDR TB). When we also removed the 33 patients still receiving treatment, the treatment success rate increased to 87.2% (87.5% for patients with MDR TB and 80% for patients with XDR TB). Patients with XDR TB were less likely to achieve sputum-smear and culture conversion (5/7, 71.4% vs. 142/177, 80.2%; p = 0.63) and required a longer duration of hospitalization (mean ± SD 202 ± 130 vs. 123.3 ± 81.0 days, p = 0.015) than patients with MDR TB. Logistic regression analysis of the association of treatment failure (death or failure) with potential covariates was performed; no statistical significant odds ratio was obtained on either the univariate or multivariate analysis; a negative prognosis related to several variables could be seen, but sample size might have influenced the statistical results (Table 2). Treatment outcomes were compared between patients who were never treated and those who were previously treated with anti-TB drugs; no statistically significant difference was evident between the 2 groups (Table 3).

**Table 2:** Logistic regression analysis of the association of treatment failure (death and failure) with potential explanatory factors*

Variables	Crude OR (95% CI)	Adjusted OR (95% CI)
Male gender	4.46 (0.56–35.5)	5.8 (0.61–56.6)
Age, y	1.05 (1.01–1.09)	1.06 (1.01–1.1)
Immigrant status	1.18 (0.13–10.2)	0.7 (0.07–6.6)
HIV seropositivity	5 (0.76–32.6)	2.5 (0.28–22.1)
Previous TB treatment >30 d	0.7 (0.26–2.2)	0.4 (0.11–1.3)
Streptomycin resistance	1.35 (0.15–11.4)	1.18 (0.13–10.69)
Ethambutol resistance	1.74 (0.52–5.7)	0.99 (0.29–3.3)
Pyrazinamide resistance	1.65 (0.57–4.7)	1.08 (0.35–3.3)
Fluoroquinolone resistance	1.67 (0.32–8.6)	0.86 (0.09–7.7)
Resistance to injectable second-line drugs	1.16 (0.3–4.5)	1.28 (0.31–5.2)
Resistance to all second-line drugs	1.18 (0.34–3.9)	1.35 (0.37–4.8)

*OR, odds ratio; CI, confidence interval; TB, tuberculosis.

**Table 3:** TB treatment outcomes in study patients not previously treated for TB compared with those treated previously for TB*

Treatment outcomes	No. (%) patients not treated previously, n = 84	No. (%) patients treated previously, n = 100	p value	95% CI
Cured	40 (47.6)	42 (42)	0.49	−0.09 to 0.19
Completed	8 (9.5)	19 (19)	0.05	−0.19 to −0.001
Successful treatment (cured + completed)	48 (57)	61 (61)	0.58	−0.18 to 0.1
Died	7 (8.3)	8 (8)	1	−0.07 to 0.07
Failure	1 (1.19)	–	–	–
Treatment failure (death or failure)	8 (9)	8 (8)	0.8	−0.07 to 0.09
Default	–	1 (1)	–	–
Transferred out	13 (15.5)	12 (12)	0.55	−0.06 to 0.12
Uncertain outcome (default + transferred out)	13 (15.5)	13 (13)	0.69	−0.08 to 0.12
Still receiving treatment	15 (17.9)	18 (18)	0.85	−0.12 to 0.1
*TB, tuberculosis; CI, confidence interval.				

Seventy-four (40.2%) of the 184 study patients were treated with linezolid. Fifty-eight (78.4%) of them were born in the FSU, and 44 (59.5%) had received previous treatment. Two (2.7%) were HIV seropositive. *M. tuberculosis* isolates of patients receiving linezolid treatment were more frequently resistant to pyrazinamide (49/74, 66.2% vs. 30/110, 27.3%; p<0.001), capreomycin (16/74, 21.6% vs. 3/110, 2.7%; p<0.001), amikacin (15/74, 20.8% vs. 8/110, 7.3; p = 0.009), fluoroquinolones (14/74, 18.9% vs. 6/110, 5.5%; p = 0.004) and cycloserine (6/74, 8.1% vs. 3/110, 2.7%; p = 0.16). Patients with XDR TB were more frequently treated with linezolid (5/7, 71.4% vs. 69/177, 38.9%; p = 0.12) than other patients with MDR TB. In the group of patients with linezolid treatment, the median time to sputum-smear conversion (XDR TB: 134 days vs. 44 days; MDR TB: 57 days vs. 36.5 days; log rank p = 0.0213) and to culture conversion (XDR TB: 160 days vs. 105 days; MDR TB: 68 days vs. 59 days; log rank p = 0.0023) was longer than in the group of patients not receiving linezolid (Figure). However, the duration of hospitalization was comparable in both groups (mean ± SD 135.4 ± 84.1 days with linezolid vs.120.5 ± 84.2 days without linezolid; p = 0.241) as was the case-fatality rate (p = 0.28). Different outcomes (e.g., successful treatment) were identified between those treated with linezolid versus those without linezolid (Table 4). Adverse effects ascribed to linezolid were observed in 25 (33.8%) of 74 cases (35% in cases with MDR and 20% in cases with XDR TB). Linezolid was interrupted in 19 (76%) of 25 cases and not reintroduced in 11 (58%) of 19 cases. Severe anemia appeared in 14 (56%) of 25 cases.

**Figure:**
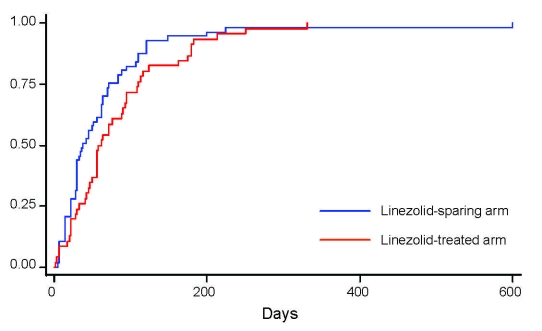
Kaplan-Meier plot showing the time to sputum smear conversion according to treatment received (linezolid-containing regimen, n = 74, vs. linezolid-sparing regimen, n = 110) in Germany (log-rank test 0.0924). The proportion of case-patients reaching conversion is shown along the vertical axis.

**Table 4:** TB treatment outcomes in study patients treated with linezolid compared with those not treated with linezolid*

Treatment outcomes	No. (%) patients treated with linezolid, n = 74	No. (%) patients not treated with linezolid, n = 110	p value	95% CI
Cured	21 (28.4)	61 (55.5)	0.0003†	−0.41 to −0.13
Completed	12 (16.2)	15 (13.6)	0.56	−0.07 to 0.13
Successful treatment (cured + completed)	33 (44.6)	76 (69.1)	0.0007†	−0.39 to −0.1
Died	8 (10.8)	7 (6.4)	0.31	−0.04 to 0.12
Failure	1 (1.4)	–	–	–
Treatment failure (death or failure)	9 (12.2)	7 (6.4)	0.15	−0.02 to 0.14
Default	–	1 (0.9)	–	–
Transferred out	10 (13.5)	15 (13.6)	0.98	−0.1 to 0.09
Uncertain outcome (default + transferred out)	10 (13.5)	16 (14.5)	0.84	−0.11 to 0.09
Still on treatment	22 (29.7)	11 (10)	0.0009†	0.07 to 0.3

*TB, tuberculosis; CI, confidence interval. †Significant result (p<0.05).

Sixty-four (36.2%) of 177 MDR TB patients showed resistance to pyrazinamide, ethambutol, or both. These patients were less likely to achieve sputum-smear and culture conversion (49 [77%] of 64 vs. 93 [82%] of 113; p = 0.36) and were more frequently treated with linezolid (38 [60%] of 64 vs. 31 [27%] of 113; p = 0.00003). Thirty (47%) of them were successfully treated, 19 (30%) were still receiving treatment, one’s treatment failed (2%), 10 (16%) were lost to follow-up, and 4 died (6%). Of 7 patients with XDR TB, 6 (86%) harbored strains that were resistant to pyrazinamide and ethambutol. Three (50%) of them achieved successful treatment outcome, 2 (33%) were still receiving treatment, and 1 (16.7%) died. These patients with XDR TB required longer hospitalization than those with MDR TB with resistance to pyrazinamide and ethambutol (mean ± SD 210.7 ± 140.1 vs. 132.5 ± 92.8 days; p = 0.063).

## Discussion

We present the results of our national survey on clinical parameters associated with MDR and XDR TB in a Western European country. Of the patients hospitalized with MDR or XDR TB in Germany who were included in this survey, 53% were treated previously against TB, and nearly 90% of them had immigrated from FSU countries. Relatively high treatment success rates were achieved with conventional medical treatment, intensified medical care, including long-term inpatient treatment, directly observed therapy, and use of third-line anti-TB drugs. Less then 6% of patients with MDR TB required a surgical intervention.

In the German observational cohort, the proportion of MDR TB among all TB cases was 4%. Strains of *M. tuberculosis* in 7 (3.8%) of 184 patients with MDR TB met the case definition for XDR TB, an infection now recognized as a global problem (*10*). Alarming reports on the spread of XDR TB among HIV-seropositive persons have been published recently for Kwa Zulu Natal, South Africa (*12*). While HIV coinfection was not a risk factor for XDR TB in our cohort, XDR TB was related to previous treatment mismanagement including the lack of directly observed therapy in FSU countries.

Patients with XDR TB have a higher risk for death and treatment failure than those with MDR TB (*21*,*22*). In infections with MDR TB, drug resistance to additional first-line drugs other than isoniazid and rifampin has recently been shown to be a predictor of negative treatment outcomes (*13*). Resistance to fluoroquinolones and injectable second-line drugs (capreomycin in particular) also contributes to increased risk for treatment failure and death in these cases (*23*,*24*). XDR TB–defining drugs are those considered essential to achieve successful outcomes in MDR TB cases (*9*,*17*,*24*,*25*). While rapid direct sensitivity testing of *M. tuberculosis* for all cases with a suspicion of multidrug resistance is highly important, this technology is currently not available in many geographic areas with a high incidence of MDR TB.

Our findings support the observation that treatment success in cases with MDR TB is dependent on the number of drugs the strain is resistant to and the previous treatment history. The probability to observe any TB drug resistance or MDR TB has been shown to be 4-fold and 10-fold higher when patients have received TB treatment in the past (*8*,*26*,*27*).

As expected, patients infected with strains of XDR TB and MDR TB resistant to all first-line drugs were more likely to have a poor treatment outcome than patients infected with other strains of multidrug-resistant *M. tuberculosis*. Patients with XDR TB required longer hospitalization and were less likely to achieve sputum-smear and culture conversion, although the latter result was not statistically significant.

More than 40% of patients in this cohort received off-label treatment against MDR or XDR TB with the oxazolidinone linezolid (*28*). In vitro and pharmacogenetic data suggest that oxazolidinones could be useful in management of mycobacterial infection, including MDR TB (*29*–*32*). However, clinical experience with the use of linezolid in the management of mycobacterial infections has been mainly restricted to case reports in nontuberculous mycobacterial diseases (*33*–*35*) and to a few case reports on patients with MDR TB (*28*,*36*,*37*). Cases of 24 patients with mycobacterial infections who were treated with linezolid were recently reviewed (*38*). Sterilization of mycobacterial cultures or resolution of symptoms was achieved in 15 (62.5%) of the 24 cases, although serious adverse events were observed in up to 75% of patients.

In this study, the description of 74 patients who were treated with linezolid against MDR or XDR TB in routine clinical practice substantially adds to the knowledge of the efficacy and tolerability of this drug. Drug toxicity from linezolid occurred in more than one third of patients and lead to treatment discontinuation in 76% of them. Patients who were treated with linezolid had a much higher level of drug resistance than those who were not treated with this drug, and they had a longer time to sputum-smear and culture conversion. Nevertheless, patients who were treated with a linezolid-containing regimen experienced a sustained culture conversion rate of almost 80%. Despite the fact that patients who were treated with linezolid had a much higher level of drug resistance, the mortality rate was comparable to that of patients with fewer drug resistances who were not treated with linezolid. Drug resistance to linezolid in cases never treated previously (occasionally reported) (*39*) was extremely low in this cohort (1/184 patients with MDR TB). These data suggest that a linezolid-containing combination treatment might be an effective option against MDR or XDR TB. However, the high frequency of adverse effects to linezolid warrants extreme caution when this drug is used for a prolonged period. Further investigations are needed to determine the best duration and dosage of linezolid treatment if the drug is to be routinely used as a life-saving therapy in cases of MDR or XDR TB.

In this cohort, most patients with MDR TB for whom complete treatment data were available were treated for a 24-month period with a combination treatment of 4 or 5 effective drugs. Long-term inpatient care (mean ± SD 202 ± 130 days for XDR TB and 123.3 ± 81.0 days for MDR TB) and availability of all third-line drugs was necessary to achieve an overall treatment success rate of 59% (overall sample) to 87% (excluding patients still on treatment and lost to follow-up) in the German TBNET hospitals. The results are consistent with those of previous studies showing overall treatment success rates in MDR TB of 54% (*13*) and 62% (*40*).

The study has several limitations. Fourteen percent of patients were lost to follow-up by their hospital physicians. Their clinical outcome is uncertain. Complete data on previous treatment regimens were not available for most patients with recurrent TB who immigrated from FSU countries. Additional factors, including variability of provider treatment practices in the patients’ native countries and existence of additional co-existing conditions, may have confounded the results of our analysis. The proportion of patients with strains of *M. tuberculosis* with more advanced drug resistance was higher among the 27 participating hospitals of the German TBNET than other hospitals in Germany, which are not specialized in TB treatment. Data for 35% of patients with MDR TB who were identified in Germany during the time of the survey were not available for this study as their cases were not diagnosed and treated in a hospital participating in this survey, which could have resulted in a selection bias. Nevertheless, the large and representative sample size, the availability of treatment outcomes, and the quality of laboratory data (all XDR TB–defining drugs tested and drug susceptibility tests controlled for quality) strengthen the results of this study.

In conclusion, cases of MDR and XDR TB in Germany appear to be largely restricted to immigrants from FSU countries. Previous treatment mismanagement is the probable cause of *M. tuberculosis* drug-resistance selection in most of these patients. Off-label treatment with linezolid is frequently used to treat advanced cases of MDR and XDR TB in Germany, despite high rates of adverse effects and paucity of clinical evidence for safety, tolerability, and efficacy of this medication. Relatively high sustained culture conversion rates can still be achieved in advanced cases of MDR and XDR TB; this requires high level, labor-intensive, and costly case management, including quality-controlled drug-susceptibility testing for all second-line drugs, long-term inpatient care, directly observed therapy, and availability of all third-line drugs. However, these resources are currently not available for patients with MDR or XDR TB in many other places outside Western Europe.

## Supplementary Material

Appendix Table Clinical characteristics among patients with XDR TB, Germany

Appendix Figure A) Distribution of countries of origin of patients with multidrug-resistant/extensively drug-resistant tuberculosis in Germany. FSU, former Soviet Union. B) Distribution of countries of origin among FSU countries
