# Supplementary material for: Multidrug- and Extensively Drug-Resistant Tuberculosis, Germany
Source: Emerg Infect Dis. 2008 Nov;14(11):1700–6. doi: 10.3201/eid1411.080729 (PMC2630755; doi:10.3201/eid1411.080729)
Supplement: Appendix Table — Clinical characteristics among patients with XDR TB, Germany [file 08-0729_appT-s1.pdf]

Publisher: CDC; Journal: Emerging Infectious Diseases
Article Type: Research; Volume: 14; Issue: 11; Year: 2008; Article ID: 08-0729
DOI: 10.3201/eid1411.080729; TOC Head: Research

Appendix Table._Clinical characteristics among patients with XDR TB, Germany

Country of birth	Age, y	Gender	Radiology at XDR diagnosis	Previous treatment, >30 d	Drugs received during previous treatment†	Drug-resistance at XDR diagnosis†	Drugs used in current treatment†	Hospital stay, d	Smear conversion, d	Culture conversion, d	Outcome	TX duration, mo
Azerbaijan	52	M	Monolateral cavity	Y	Unknown	Am, Cm, Cs, E, FQ, H, PAS, R, Rb, S, Z	Eto, Lzd + surgery	224	7	35	Died	24
China	33	F	Generalized lymphadenopathy	N	NA	Am, Cm, E, FQ, H, R, S, Z	Cs, Eto, Rb,	59	44	93	TX completed	26
Uzbekistan	33	M	Monolateral cavity	Y	E, H, R, S,Z	Am, Cm, E, Eto, FQ, H, R, Rb, S, Z	Cm, Cs, Lzd,	120	Not achieved	Not achieved	Chronic in treatment	17
Uzbekistan	29	M	Bilateral cavities	Y	E, H, R, Rb S, Z	Am, Cm, E, FQ, H, R, Rb, S, Z	Cs, Eto, Lzd, Rb	240	180	160	TX completed	12
Russian Federation	60	M	Monolateral cavity	Y	E, H, R,Z,	Am, E, FQ, H, R, Rb, S	Cm, Eto , FQ, PAS, Z	150	Unknown	117	TX completed	24
Ukraine	50	M	Bilateral cavities	Y	Unknown	Cm, E, Eto, FQ, H, R, Rb S, Z	Am, Cs, Lzd, PAS	159	88	Not achieved	Chronic in treatment	15
Ukraine	42	M	Bilateral cavities	Y	Unknown	Am, Cm, E, Eto, FQ, H, R, Rb, Z	Cs, Lzd, PAS, S	462	330	300	TX completed	15

*XDR TB, extensively-drug resistant tuberculosis; TX, treatment; Y, yes; N, no; NA, not applicable.
†First-line drugs; H, isoniazid; E, ethambutol; R, rifampin; S, streptomycin; Z, pyrazinamide. Second- and third-line drugs: Am, amikacin; Cm, capreomycin; Cs, cycloserine; Eto, ethionamide; FQ, fluoroquinolone; Lzd, linezolid; PAS, para-aminosalycilic acid; Rb, rifabutin.
