# Supplementary figures and images for: Multidrug- and Extensively Drug-Resistant Tuberculosis, Germany
Source: Emerg Infect Dis. 2008 Nov;14(11):1700–6. doi: 10.3201/eid1411.080729 (PMC2630755; doi:10.3201/eid1411.080729)

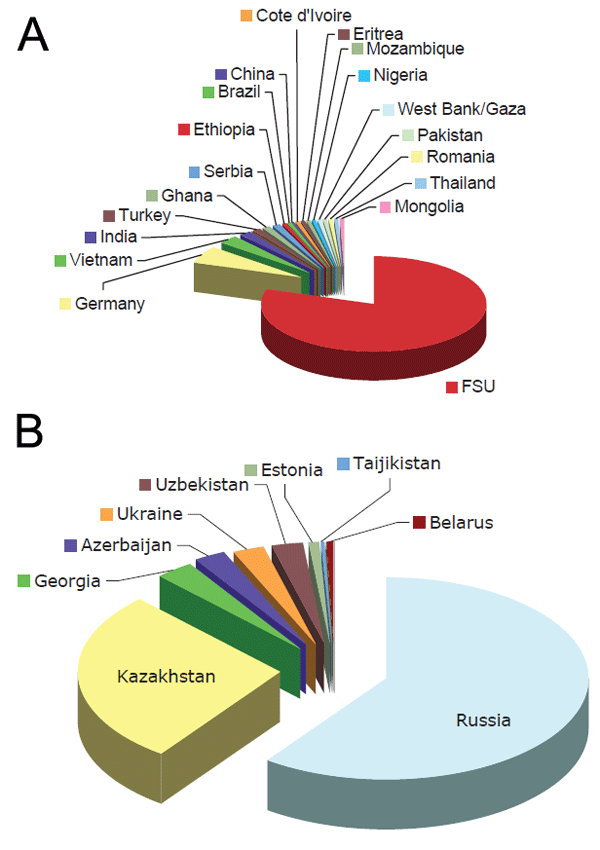

Supplement: Appendix Figure — A) Distribution of countries of origin of patients with multidrug-resistant/extensively drug-resistant tuberculosis in Germany. FSU, former Soviet Union. B) Distribution of countries of origin among FSU countries [file 08-0729_app-s2.gif]
